# Prevalence of adverse drug reactions in the primary care setting: A systematic review and meta-analysis

**DOI:** 10.1371/journal.pone.0252161

**Published:** 2021-05-26

**Authors:** Widya N. Insani, Cate Whittlesea, Hassan Alwafi, Kenneth K. C. Man, Sarah Chapman, Li Wei

**Affiliations:** 1 Research Department of Practice and Policy, School of Pharmacy, University College London, London, United Kingdom; 2 Department of Pharmacology and Clinical Pharmacy, Center of Excellence for Pharmaceutical Care Innovation, Padjadjaran University, Bandung, Indonesia; 3 Faculty of Medicine, Umm Al Qura University, Mecca, Saudi Arabia; 4 Department of Pharmacology and Pharmacy, University of Hong Kong, Hong Kong, Hong Kong; 5 Department of Pharmacy and Pharmacology, University of Bath, Bath, United Kingdom; Nord University, NORWAY

## Abstract

**Background:**

Adverse drug reactions (ADRs) represent a major cause of iatrogenic morbidity and mortality in patient care. While a substantial body of work has been undertaken to characterise ADRs in the hospital setting, the overall burden of ADRs in the primary care remains unclear.

**Objectives:**

To investigate the prevalence of ADRs in the primary care setting and factors affecting the heterogeneity of the estimates.

**Methods:**

Studies were identified through searching of Medline, Embase, CINAHL and IPA databases. We included observational studies that reported information on the prevalence of ADRs in patients receiving primary care. Disease and treatment specific studies were excluded. Quality of the included studies were assessed using Smyth ADRs adapted scale. A random-effects model was used to calculate the pooled estimate. Potential source of heterogeneity, including age groups, ADRs definitions, ADRs detection methods, study setting, quality of the studies, and sample size, were investigated using sub-group analysis and meta-regression.

**Results:**

Thirty-three studies with a total study population of 1,568,164 individuals were included. The pooled prevalence of ADRs in the primary care setting was 8.32% (95% CI, 7.82, 8.83). The percentage of preventable ADRs ranged from 12.35–37.96%, with the pooled estimate of 22.96% (95% CI, 7.82, 38.09). Cardiovascular system drugs were the most commonly implicated medication class. Methods of ADRs detection, age group, setting, and sample size contributed significantly to the heterogeneity of the estimates.

**Conclusion:**

ADRs constitute a significant health problem in the primary care setting. Further research should focus on examining whether ADRs affect subsequent clinical outcomes, particularly in high-risk therapeutic areas. This information may better inform strategies to reduce the burden of ADRs in the primary care setting.

## Introduction

Adverse drug reactions (ADRs) represent a significant clinical problem in healthcare, owing to the increasing multimorbidity and complexity of medical treatment. ADRs are defined as "noxious and unintended responses to a medicinal product" [1]. Since 2010, this definition has included reactions not only from appropriate use of drugs at normal doses, but also those resulted from errors and the use outside the term of authorization [2]. Lazarou et al estimated from a meta-analysis, that ADRs represent the fourth leading causes of death in the United States (US) [3]. In England, Hospital Episode Statistic (HES) data showed that between 2008 and 2015, there were 541,416 hospital admissions caused by ADRs, representing 1.5% of total hospital episodes; over this period the number of ADRs-related hospital admissions increased by 53.4% [4, 5].

While a substantial body of work had been undertaken to characterise ADRs that resulted in hospital admissions and occurred during hospital stay [6–11], much less is known about the overall burden of ADRs in the primary care setting, where most medications are prescribed and administered [12]. Identification of ADRs in the primary care setting is inherently challenging due to the intermittent nature of healthcare contacts and scattered information across multiple patient care providers [13]. As a gatekeeper, primary care provider has a critical role in signalling and recognising ADRs to minimise the subsequent impact of the reaction and ensure optimal individual pharmacotherapy [14].

Previous systematic reviews have been conducted in primary care setting, but these reviews focused on medication errors [15] and general safety incidents, e.g., diagnostic incidents, administrative and communication incidents, and medication management incidents [16]. Tache et al examined medication-related adverse events, but the review combined both primary and secondary care settings and included six ambulatory-based studies only up to 2011 [13]. Another review has been conducted by Khalil et al, however no meta-analysis, evaluation of study quality, heterogeneity analysis, and preventability assessment were performed [17]. Ascertaining the burden of ADRs in the community has significant public health implication, as this information may help in prioritising areas of improvement, and thus potentially decreasing patients’ risk of untoward therapeutic consequences. Therefore, this systematic review and meta-analysis were performed to investigate the prevalence of ADRs in the primary care setting, their preventability, and factors affecting the heterogeneity of the estimates.

## Methods

The Preferred Reporting Items for Systematic Reviews and Meta-Analyses (PRISMA) statement was used to guide the reporting of the findings. A completed PRISMA checklist is provided as an additional file (S1 Appendix). The study protocol was registered in the International Prospective Register of Systematic Reviews database (PROSPERO: CRD 42020191159).

### Search strategy

A systematic search was conducted within Medline, Embase, Cumulative Index of Nursing and Allied Health Literature (CINAHL), and International Pharmaceutical Abstracts (IPA) databases across all publication dates up to June 2020. The search strategies cover the terms related to ADRs and setting of interest (S2 Appendix). The reference lists of eligible studies were reviewed to identify potential relevant studies. The corresponding authors of the eligible articles were contacted when additional information was needed.

### Eligibility criteria

1. Study type: Observational studies that provided information on the prevalence, i.e., the rate of patients with ADR(s) within the observed period were included. Studies that reported the occurence of ADRs in relation to total consultations or total course of drug therapies without reporting the number of patients with ADR(s) and total number of patients included, were not eligible for inclusion to ensure comparability of outcome measure.
2. Population and setting: Patients from all age groups receiving care from primary care facilities were included. Primary care is defined as the first point of contact with healthcare system, providing generalist care delivered outside inpatient setting [16, 18]. This setting included general/family medicine, general internal medicine, general paediatrics, community pharmacy, and community health services such as long-term care facilities [16]. As primary care practitioners are commonly responsible for the provision of first-line health care to long-term care facilities residents [19, 20], we included studies investigating ADRs in long-term care facilities. General internal medicine was included only when the studies specified that they provided primary care services for the patients, as typically observed in the context of US primary care health system [21].
3. Types of outcome: The outcome of interest was ADRs, defined as "noxious and unintended responses to a medicinal product" [1]. For example, muscle symptoms/myopathy associated with statin, cough associated with angiotensin converting enzyme inhibitor (ACEI), and ankle oedema associated with calcium channel blocker (CCB). Since 2010, this definition has included reactions not only from appropriate use of drugs at normal doses, but also those resulted from errors at any medication process [2], e.g., myopathy in a statin user who was previously prescribed systemic azole antifungal and rash after admistration of flucloxacillin in a patient with a documented allergy to penicillin [22, 23].

The eligible detection methods were one or a combination of the following [24];

1. Spontaneous/solicited reporting by healthcare professionals, which involves active participation of clinicians to collect and notify any ADRs observed during primary care consultations to research investigators within a specified period of time [25, 26].
2. Medical record/notes/medication review, either using prospective or retrospective review. This method could be combined with patient survey [23, 27]. We included studies using medical record review alone or combined record/medication review-patient survey.
3. Trigger-based medical record review, which involves a two-step review process [28, 29]. Firstly, a selection of patient record was screened using a set of pre-defined ADRs triggers, e.g., specific laboratory values, prescribing of antidote medication, specific phrases, or drug-event potentially indicative of ADRs. For example, on warfarin treatment and international normalised ratio (INR) > 5, on statin treatment and serum aspartate amino transferase (AST) > 150 U/L; and on diuretics treatment and serum potassium < 3.0 mmol/L [30, 31]. Subsequently, the investigators performed thorough reviews of these flagged charts to determine whether the use of drug was associated with the event or ADRs had actually occurred [28, 29, 32, 33].
4. Administrative database screening to identify ADRs recorded by primary care providers during routine care. These reactions were typically recorded using specific designated codes for ADRs, e.g., International Classification of Primary Care (ICPC) Code A-85 or Read Code Chapter TJ [14, 34].

### Exclusion criteria

Studies investigating ADRs as causes of emergency department visits and/or hospital admission were excluded. Studies with combined setting that did not provide separate estimate of ADRs between primary and secondary/tertiary care setting were excluded. Studies that assessed ADRs using only public surveys without any further assessment by healthcare professional/research investigator were excluded to ensure comparability of outcome measure. Studies that examined ADRs associated with specific drug exposure were excluded as the samples were not generalizable of primary care population in general. Literature review, cases reports/series, and conference abstracts were excluded, as were articles written in languages other than English.

### Screening and data extraction

Two investigators (WI and HA) independently screened the titles and abstracts generated from the databases using the predetermined criteria. Any discrepancies between the two reviewers were resolved through discussion. Following initial screening, the full-text of potentially relevant papers were further assessed to identify eligible studies. The process of study selection was presented using an adapted PRISMA diagram [35]. The process of data extraction was conducted using a standardized data collection form for all included studies. Data extracted included general characteristics of the studies, ADRs prevalence, and when reported: drugs implicated in the ADRs, preventability, severity, and risk factors of ADRs.

### Appraisal of study quality

The quality of the included studies were examined using Smyth ADRs adapted scale [36]. This 10-item instrument was developed specifically for studies examining ADRs in clinical settings [37, 38]. The following aspects were evaluated from each study; study design, data source, methods of ADRs detection, assessment of causality, preventability, and severity [36].

### Data analysis

A random-effects model was used to calculate the pooled prevalence of ADRs and the percentage of preventable ADRs. Heterogeneity among the included studies was assessed using I^2^ statistics. Sub-group analyses and meta-regression were performed to explore potential source of heterogeneity, i.e., age groups, ADRs detection methods, ADRs definitions, setting, study quality, and sample size. All analyses were performed in Stata version 15.

## Results

### Literature search and selection process

**Fig 1:**
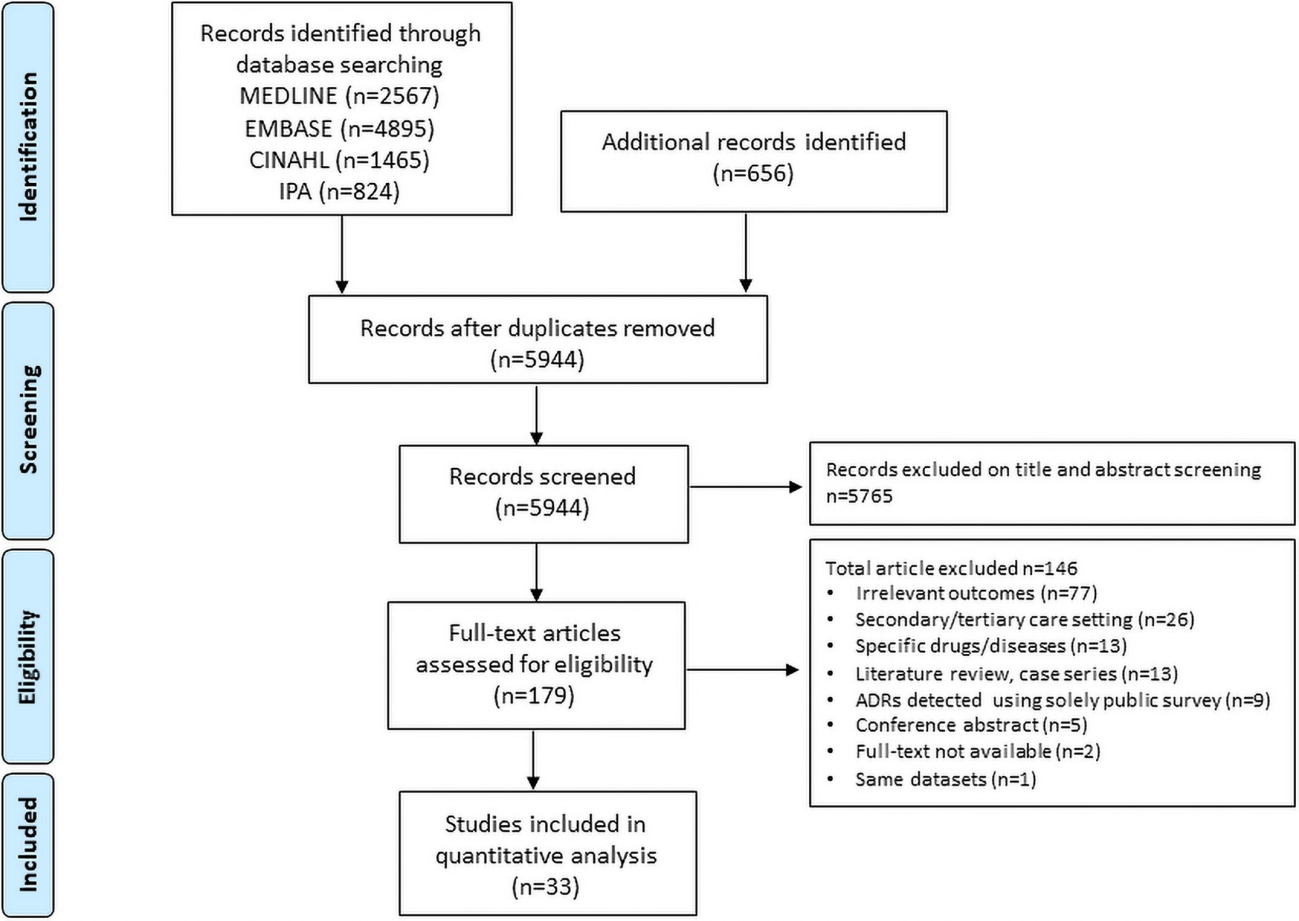
Flow diagram of the selection of eligible studies.

A total of 10,407 citations were retrieved from the electronic databases and other sources. After removal of duplicates, 5944 records remained for evaluation. Title and abstract screening yielded 179 records eligible for full-text assessment. Finally, a total of 33 studies were included in this systematic review (Fig 1) (Table 1).

**Table 1:** General characteristics of included studies.

Reference	Country	Setting	Method for ADRs Detection	Mean age ± SD; range	Sample Size	ADRs Definition /Causality	Prevalence (95% CI)
*Adults population*							
Schneider et al, 1992 [39]	United States	Primary Care Internal Medicine	Medical record review	77.2 ± 5.6; elderly, 58–97 years	463	WHO^a^/Naranjo algorithm	20.95 (17.24, 24.66)^b^
Montastruc et al, 1995 [25]	France	General Practice	Solicited reporting by physicians	49.2 ± 24.7; 17–85 years	2094	WHO/French causality method	1.00 (0.58, 1.41)^b^
Cooper et al, 1996 [40]	United States	Long-term Care Facilities	Medication review and direct patient assessment	80.6 ± NA; elderly, range was not specified	332	WHO/Naranjo algorithm	65.36 (60.24, 70.47)^b^
Hanlon et al, 1997 [41]	United States	Primary Care Internal Medicine	Medical record review and patient survey	69.4 ± 3.5; elderly, ≥ 65 years	167	WHO/NA	34.73 (27.51, 41.95)^b^
Veehof et al, 1999 [42]	The Netherlands	General Practice	Administrative database screening	71.6 ± NA; elderly,≥ 65 years	2185	NA/NA	8.92 (7.73, 10.12)^b^
Gandhi et al, 2000 [43]	United States	Primary Care Internal Medicine	Medical record review^c^	45.8 ± NA; 20–75 years	2248	Bates et al/Naranjo algorithm	2.85 (2.16, 3.53)^b^
Aspinall et al, 2002 [44]	United States	Primary Care Internal Medicine	Medical record review and patient survey	68.0 ± 10.5; range was not specified	198	WHO/Naranjo algorithm	25.76 (19.67, 31.85)^b^
Gandhi et al, 2003 [23]	United States	Primary Care Internal Medicine	Medical record review and patient survey	52.0 ± NA; 19–100 years	661	Bates et al/Defined by authors	24.51 (21.23, 27.79)^b^
Roughead et al, 2004 [45]	Australia	Home Setting	Domiciliary medication review and survey^c^	men: 74.0, women: 75.5 years^d^ ± NA; elderly, range was not specified	1000	NA/NA	18.60 (16.19, 21.01)^b^
Sorensen et al, 2005 [46]	Australia	Home Setting	Domiciliary medication review and survey	72.4 ± 10.3; 37–99 years	204	NA/NA	25.00 (19.05, 30.94)^b^
Nguyen et al, 2006 [47]	United States	Long-term Care Facilities	Voluntary reporting by healthcare professional and trigger-based medical record review	72.0 ± NA; elderly, 65–100 years	335	WHO/Naranjo algorithm	61.79 (56.58, 66.94)^b^
Calderon-Larranaga et al, 2012 [48]	Spain	General Practice	Administrative database screening	NA; ≥ 14 years	79,089	NA/NA	0.87 (0.81, 0.94)^b^
Brenner et al, 2012 [30]	United States	Primary Care Internal Medicine	Trigger-based medical record review	55.0 ± 14.0; > 18 years	516	Bates et al/NA	17.64 (14.35–20.92)^b^
Miller et al, 2013 [49]	Australia	General Practice	Solicited reporting by physicians^c^	NA; ≥ 45 years	7518	Britt et al/NA	10.79 (10.09, 11.49)^b^
Sino et al, 2013 [50]	The Netherlands	Home Setting	Medication review and interview	79.3 ± NA; ≥ 45 years	115	NA/Defined by authors	40.00 (31.04, 53.91)^b^
Marcum et al, 2013 [51]	United States	Long-term Care Facilities	Trigger-based medical record review	70.6 ± 12.2; elderly, range was not specified	321	Bates et al/NA	20.25 (15.85, 24.64)^b^
Eguale et al, 2015 [52]	Canada	General Practice	Administrative database screening	NA; ≥ 18 years	46,021	NA/NA	7.57 (7.33, 7.81)^b^
Rhalimi et al, 2017 [53]	France	Community Pharmacy	Medication review and survey^c^^,^^e^	80.6 ± 6.6; elderly, ≥ 65 years	892	WHO/NA	3.36 (2.18, 4.55)^b^
Devik et al, 2018 [54]	Norwegia	Long-term Care Facilities and Home Care	Medication review^c^	87.0 ± NA; 65–102 years	154	NA/NA	21.43 (14.94, 27.90)^b^
Benson et al, 2018 [55]	Australia	General Practice	Medication review and patient survey^c^	67.7 ± 13.6; range was not specified	493	NA/NA	11.15 (8.37, 13.93)^b^
Cahir et al, 2019 [56]	Ireland	General Practice	Medical record review and patient survey	NA; elderly, ≥ 70 years	859	Parry et al/NA	24.0 (23.0, 25.0)
Sell et al, 2020 [57]	Germany	Community Pharmacy	Medication review and patient survey^c^	72.0 ± NA; range was not specified	1090	NA/NA	21.19 (18.77, 23.62)^b^
*Paediatric population*							
Horen et al, 2002 [26]	France	Paediatric Practice	Solicited reporting by physicians	NA; ≤ 16 years	1419	NA/French causality method	1.41 (0.80, 2.02)^b^
Jonville et al, 2002 [58]	France	Paediatric Practice	Solicited reporting by physicians	NA; paediatric, range was not specified	1192	NA/French causality method	0.67 (0.21, 1.13)^b^
*All age groups*							
Honigman et al, 2001 [31]	United States	Multidisciplinary	Computerised trigger-rules record review	47.9 ± NA; all (<31 to >75 years)	15,665	Bates et al/Naranjo algorithm	5.52 5.16, 5.87)^b^
Miller *et al*, 2006 [59]	Australia	General Practice	Solicited reporting by physicians^c^	NA; all (<1 to >75 years)	8215	Britt et al/NA	9.88 (9.24, 10.53)^b^
Lewinski et al, 2010 [60]	Germany	Community Pharmacy	Medication review and patient survey^c^	50.4; all (<16 to >64 years)	3040	NA/NA	5.70 (4.88, 6.52)^b^
Gonzalez-Rubio et al, 2011 [61]	Spain	General Practice	Administrative database screening	NA; all (0 to ≥ 76 years)	126,838	WHO/NA	0.43 (0.39, 0.46)^b^
Frokjaer et al, 2012 [62]	Denmark	Community Pharmacy	Medication review and patient survey^c^	NA; all (0 to > 65 years)	3868	NA/NA	2.90 (2.37, 3.43)^b^
Trinkley et al, 2017 [27]	United States	Primary Care Internal Medicine	Medical record review and patient survey	52 ± 16; 7–95 years	701	Bates et al/Naranjo algorithm	10.84 (8.54, 13.14)^b^
Iancu et al, 2015 [63]	Romania	Community Pharmacy	Medication review and patient survey^c^	NA; range was not specified	3155	NA/NA	3.17 (2.56, 3.78)
Hoon et al, 2017 [14]	The Netherlands	General Practice	Administrative database screening	40.7 ± NA; all (0 to >85 years)	1,256,024	WHO/NA	0.66 (0.65, 0.68)^b^
Latif et al, 2018 [64]	United Kingdom (UK)	Home Setting	Domiciliary medication review and patient survey^c^	NA; all (<24 to >75 years)	1092	NA/NA	16.80 (14.60, 19.00)

^a^WHO: World Health Organization

^b^CI was not presented in the article, but calculated from sample size and prevalence estimate.

^c^Only data on ADR was included in the analysis. Drug complications, prescribing errors, and other drug-related problems were excluded.

^d^ Median

^e^Additional data were obtained through contact with author.

### Characteristics of included studies

Majority of the included studies were cross-sectional in design [14, 25, 26, 30, 31, 39–46, 48–53, 55–64], with two retrospective cohort [47, 56] and two prospective cohort studies [23, 27]. Study periods spanned from 1992 to 2020. Almost half of the included studies were conducted in Europe (n = 16) [14, 25, 26, 42, 48, 50, 53, 54, 56–58, 60–64], about one-third in North America (n = 12) [23, 27, 30, 31, 39–41, 43, 44, 47, 51, 52] and five in Australia [45, 46, 49, 55, 59]. Majority of the studies (n = 22) focused on adult, with ten of them were performed among the elderly [39–42, 46, 47, 51, 53, 54, 56]. Nine studies were conducted among all age groups [14, 27, 31, 59–64], while the remaining two studies examined ADRs in a paediatric population [26, 58].

About one-third of the studies were performed in a general practice setting [14, 25, 42, 48, 49, 52, 55, 56, 59, 61], while seven studies were conducted within primary care internal medicine [23, 27, 30, 39, 41, 43, 44]. The remaining studies were performed in the community pharmacy (n = 5) [53, 57, 60, 62, 63], long-term care facilities (n = 4) [40, 47, 51, 54], paediatric practice (n = 2) [26, 58], and home setting (n = 4) [45, 46, 50, 64], where healthcare professionals performed domiciliary medication review.

Majority of the studies (n = 21) used medical record/notes/medication review to identify ADRs. Most of these studies combined this method with patient survey or direct patient assessment (n = 16), with two studies used telephone-based survey [23, 27]. Three studies applied trigger-based medical record review, with one study combined it with spontaneous (voluntary) reporting by healthcare professionals [30, 31, 47, 51]. Solicited reporting method were used in five studies, in which healthcare professionals were asked to notify ADRs within a specified period, ranging from a 1-week to a 3-month period [25, 26, 49, 58, 59]. The remaining five studies used administrative database screening to identify ADRs data recorded by primary care providers during routine care [14, 42, 48, 52, 61] (Table 1).

### Prevalence of ADRs

**Fig 2:**
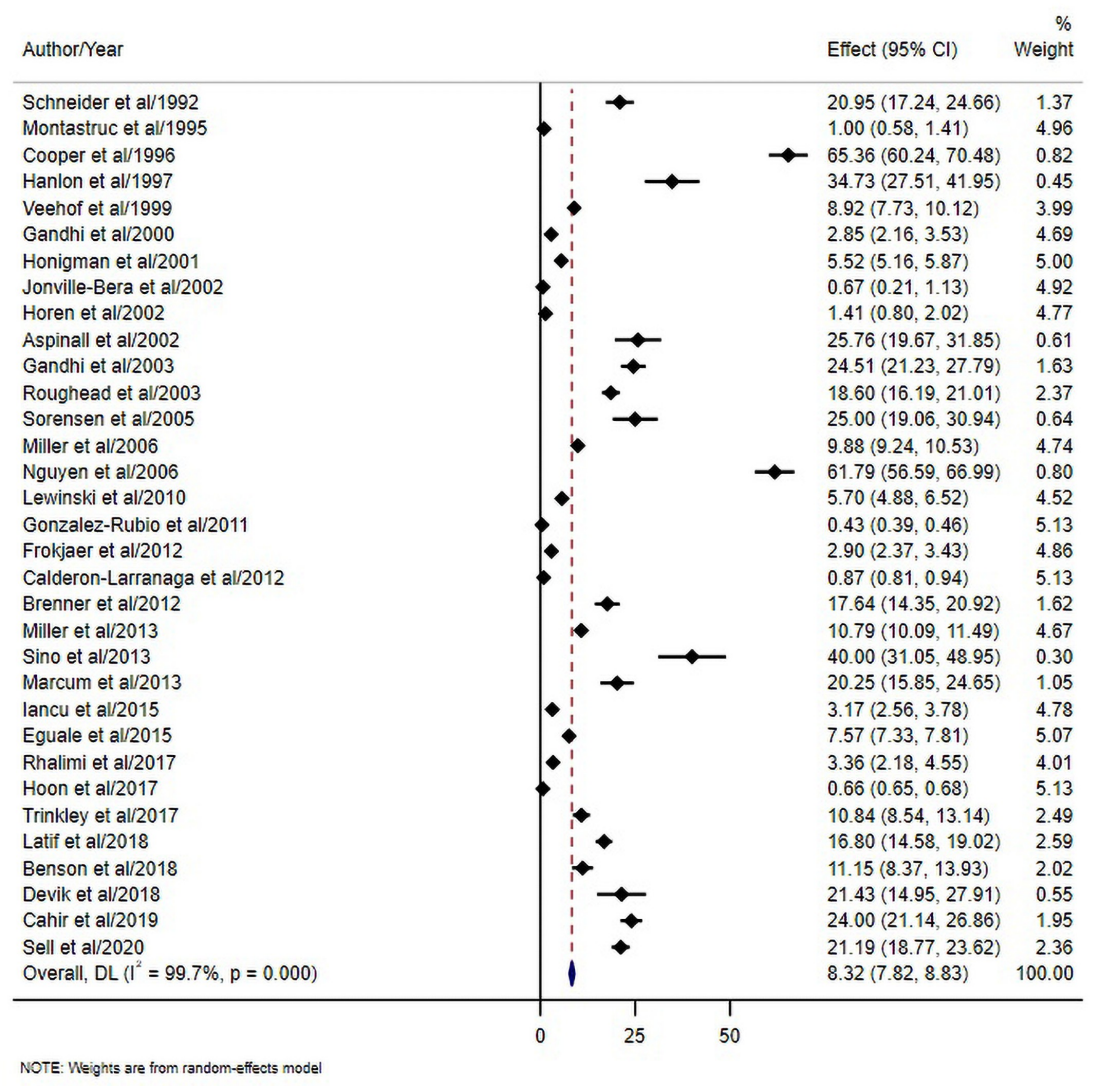
Prevalence of ADRs in the primary care setting.

The pooled estimate of ADRs among 1,568,164 individuals was 8.32% (95% CI 7.82, 8.83) (I^2^ = 99.7%) (Fig 2). When only studies with low risk of bias were considered (scored ≥ 7 in the ADRs risk of bias assessment, n = 12), the estimate increased to 20.37% (95% CI 16.89, 23.85) but the heterogeneity remains high (I^2^ = 99.5).

### Preventability of ADRs

**Fig 3:**
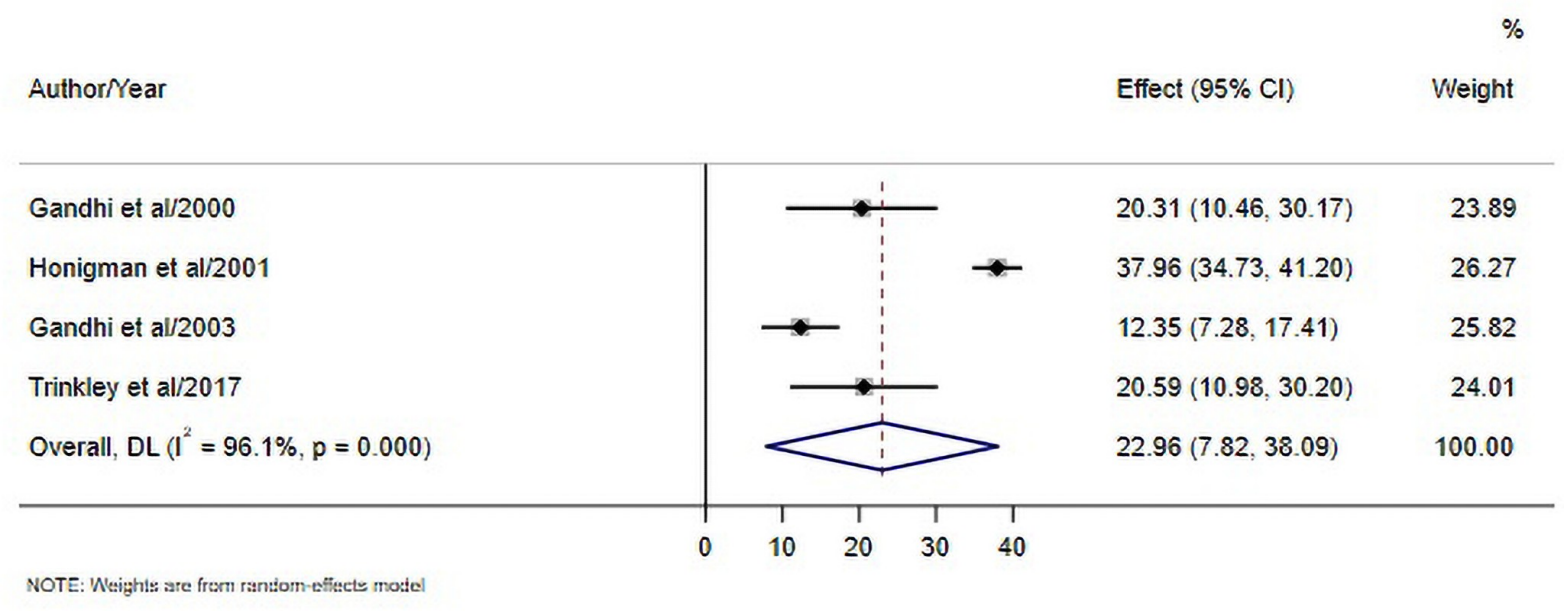
Percentage of preventable ADRs in the primary care setting.

The percentage of preventable ADRs in the primary care ranged from 12.35–37.96% [23, 27, 31, 43], with the pooled estimate of 22.96% (95% CI, 7.82, 38.09). Three studies defined preventable ADRs as reactions which due to errors in any medication process [23, 27, 31]. For example, myopathy was detected in a statin user who was recently prescribed systemic azole antifungal. Errors in acknowledging this potentially harmful drug-drug interaction during the prescribing stage led to this reaction. Thus, this myopathy was considered preventable ADR [23, 65]. One study defined preventable ADRs as reactions that occurred among patients who previously had a documented allergic reaction to the drug, and reactions which related to inadequate monitoring of the causative drug. For example, bleeding in warfarin users is considered preventable when adequate INR monitoring is not performed for patients starting warfarin [43, 66] (Fig 3). Examples of preventable and non-preventable ADRs are provided in Table 2.

**Table 2:** Example of preventable and non-preventable adverse drug reactions.

Preventability Criteria	Example	
	Preventable ADR	Non-Preventable ADR
Reaction occurred in a patient who previously had a documented prior allergic/reaction to the current causative drug.	Rash developed after administration of flucloxacillin in a patient with a documented allergy to penicillins [23, 43].	Rash developed after administration of flucloxacillin in a patient with no documented allergy to penicillins.
Reaction occurred due to any errors in medication process, including error during prescribing, dispensing, and administration.	Myopathy developed in a statin user who was recently prescribed systemic azole antifungal (Fail to identify drug-drug interaction) [23, 65].	Reaction developed without indication of possible drug-drug interaction.
	Exacerbations of asthma related with NSAID use (Contraindication overlooked) [67].	Reaction occurred despite appropriate selection of medication for patients’ condition.
	Rectal haemorrhage in a dabigatran user >75 years old related with incorrect dosage. Patient was given maximum recommended dosage (220mg/day), while in the SmPC of dabigatran, patient ≥ 75 years should receive lower dose (150 mg/day) [68]	Reaction occurred despite appropriate selection of dosage for patients’ condition.
	Hypoglycemic event due to medication administration error [69].	Reaction occurred despite appropriate dosing administration.
Required therapeutic drug monitoring or other necessary laboratory tests were not (adequately) performed.	Bleeding occurred in warfarin user with inadequate frequency of INR monitoring (led to elevated INR, e.g., INR > 4.5) [66].	Bleeding occurred despite the target INR had been achieved.
	Symptomatic hyperkalaemia in spironolactone user related with inadequate potassium monitoring [70]	Reaction occurred despite adequate laboratory monitoring and regimen adjustment.

NSAID: non-steroidal antiinflammatory drugs. SmPC: summary of product characteristic.

### Severity of ADRs

One-third of the included studies (n = 11) assessed the severity of the ADRs. The criteria used to classify severity varied between studies. Mild reactions were typically defined as reactions which did not require; i.) change in drug regimen, and ii.) specific antidote/treatment for the reactions. Moderate reactions are those requiring change in drug regimen and/or specific antidote/treatment to relieve ADRs; limits daily activities. Severe ADRs were potentially life-threatening reactions, require hospitalization, and result in significant disability [23, 26, 27, 31, 40, 43, 44, 47, 49, 56, 59]. Based on the included studies, the majority (76.0–96.3%) of ADRs in primary care were of mild-moderate severity, for example drug rash, easily bruising and bleeding related with aspirin which did not require hospitalization, indigestion/heartburn related with anti inflammatory and antirheumatic drug, dizziness/lightheadedness related with beta-blocker, sexual dysfunction related with selective serotonin reuptake inhibitor (SSRI) and beta-blocker, cough and orthostatic hypotension related with ACEI, muscle symptom related with statin, ankle swelling related with CCB, and throat pain related with oral bisphosphonate [23, 26, 27, 31, 40, 43, 44, 47, 49, 56, 59]. Up to 62.8% of the reactions required changes in drug regimen. About 1.35–9.1% of the reactions required visits to emergency department and/or hospital admission, for example bradycardia related with beta-blocker and hypoglycemic event related with sulfonylureas. Half of the patients with ADRs reported interferences with work, leisure, or daily activities; and anxiety/discomfort [23, 26, 27, 31, 40, 43, 44, 47, 49, 56, 59].

### Subgroup analysis and meta-regression

We performed subgroup analysis to investigate how the prevalence estimate varied across different subgroup of studies and potential source of heterogeneity. The analysis was performed through stratification by age group, methods to identify ADRs, definition, setting, risk of bias, and sample size. We found that studies performed among the elderly (≥ 65 years) showed the highest prevalence of ADRs, with more than a quarter of these patients potentially having experienced ADRs (28.43%, 95% CI 18.65, 38.21). There was a significant heterogeneity in every age group (I^2^>99.2%), except studies among paediatric populations (I^2^ = 71.8%) with moderate heterogeneity. High heterogeneity was still observed among studies that used the same methods to identify ADRs (I^2^>97.9%), as were studies using the same ADRs definition (I^2^>98.3%). Studies using combined medical record/notes/medication review and patient survey (n = 16) exhibited the highest prevalence (19.92%, 95% CI 16.11, 23.73). Studies which applied the WHO definition [1] (n = 9) had lower estimates compared to Bates et al definition [22, 71] (n = 6) with the prevalence of 13.05% (95% CI, 9.37, 16.73). With regard to the study setting, the prevalence of ADRs in studies conducted in long-term care facilities were higher than other units, with 42.22% (95% CI 17.57, 66.88) of the residents potentially experiencing ADRs. A large difference was observed among studies involving different sample sizes (i.e., 0–1000, 1001–10,000, and >10,000), with studies having a larger sample size tending to have a lower prevalence of ADRs. Factors affecting heterogeneity of the prevalence were further assessed using meta-regression. There were significantly higher estimates of prevalence of ADRs in studies using different ADRs detection methods, age group, setting, and sample size (P<0.05) (Table 3).

**Table 3:** Subgroup analysis of included studies.

Study Characteristics	Subgroup analysis			Meta Regression	
	Studies (n)	Pooled estimate (%) (95% CI)	I^2^ (%)	Mean Difference	P-Value
**Age groups**					
Paediatric	2	1.01 (0.29, 1.73)	71.8	ref^a^	ref
Adults (excl. elderly)	12	13.60 (10.79, 16.42)	99.7	14.56 (-6.60, 35.80)	0.170
Elderly	10	28.43 (18.65, 38.21)	99.2	27.71 (5.86, 49.56)	**0.015**
All age groups	9	4.53 (4.04, 5.03)	99.6	5.16 (-16.63, 26.95)	0.632
**Setting**					
General practice	10	5.57 (4.95, 6.19)	99.8	4.96 (-10.19, 20.11)	0.508
General internal medicine	7	19.33 (10.83, 27.84)	98.6	18.29 (-0.60, 37.18)	0.057
Community pharmacy	5	6.93 (4.10, 9.75)	98.3	6.19 (-13.45, 25.84)	0.523
Home setting	4	23.34 (17.60, 29.08)	89.6	23.55 (3.01, 44.07)	**0.026**
Long-term care facilities^b^	4	42.22 (17.57, 66.88)	98.9	39.75 (21.94, 57.55)	**0.000**
Paediatric practice	2	1.01 (0.29, 1.73)	71.8	Ref	ref
Multidisciplinary	1	-	-	-	-
**Methods**					
Medical record/notes/medication review and patient survey	16	19.92 (16.11, 23.73)	99.1	23.69 (6.49, 40.88)	**0.009**
Medical record review	3	14.90 (0.29, 29.50)	98.3	13.81 (0.66, 26.96)	**0.040**
Trigger-based medical record review	3	14.32 (3.80, 24.84)	97.9	10.69 (-7.94, 29.33)	0.249
Spontaneous/solicited reporting	5	4.74 (0.66, 8.82)	98.3	1.06 (-14.99, 17.11)	0.893
Administrative database screening	5	3.19 (2.53, 3.85)	99.9	Ref	ref
Combined	1	-	-	-	-
**Definitions**					
WHO	9	3.38 (2.81, 3.96)	99.5	Ref	ref
Bates *et al*	6	13.05 (9.37, 16.73)	98.3	-9.92 (-26.98, 7.15)	0.245
NA/Other^c^	18	11.42 (9.23, 13.60)	99.7	-10.86 (-24.10, 2.37)	0.104
**Study Quality**					
Low risk of bias	12	20.37 (16.89, 23.85)	99.5	9.47 (-2.09, 21.02)	0.105
High risk of bias	21	6.40 (5.89, 6.91)	99.6	Ref	ref
**Sample Size**					
≤1000	19	22.49 (18.58, 26.39)	99.3	20.57 (6.35, 34.79)	**0.006**
1001–10,000	9	7.67 (4.85, 10.50)	99.2	5.11 (-10.25, 20.47)	0.502
>10,000	5	2.96 (2.28, 3.64)	99.9	Ref	ref

^a^Reference value

^b^Include one studies combining nursing home and home nursing care

^c^Include three studies that used definition by Parry et al and Britt et al, the remaining studies did not specify the definition used.

### Drugs associated with ADRs

Table 4 shows information on the most common drugs class implicated in ADRs in the primary care setting. The most frequent drug class involved in the ADRs among adults were cardiovascular drugs (median 27.3%; range: 18.1–71.9%), including antihypertensive, lipid-modifying, antithrombotic drugs; followed by nervous system drugs (median 13.4%; range: 3.5–39.6%), including antidepressants, antipsychotics, analgesics; and musculoskeletal system drugs (median 8.3%; range 3.8–13.4%), including NSAIDs, antirheumatic drugs, and drugs for bone structures and mineralisation (e.g., bisphosphonates). For all age groups, the most commonly involved drugs were cardiovascular drugs (median 38%; range:23.4–73.5%), nervous system drugs (median 16.5%; range: 9.9–23.2%), and anti infectives (median 14.5%; range:8.3–20.6%). The most commonly involved drugs in the ADRs among paediatric patients were anti infectives. (median 85%; range 70–100%) [23, 26, 31, 39, 40, 42, 45, 47, 49, 51, 58, 61, 63] (Table 4).

**Table 4:** The most common drug class implicated in the ADRs in the primary care setting.

Reference	Cardiovascular system^a^	Nervous system^b^	Antiinfective^c^	Musculo-skeletal system^d^	Alimentary tract and metabolism^e^	Respiratory^f^	Hormonal system^g^
*Adults*							
Schneider et al, 1992	33/107(31.0%)	11/107 (10.2%)	-	11/107 (10.2%)	-	-	-
Cooper et al, 1996^h^	199/485 (41.0%)^i^	159/485 (32.8%)	22/485 (4.5%)	42/485 (8.6%)	-	25/485 (5.1%)	41/485 (8.4%)
Veehof et al, 1999	39/215 (18.1%)	12/215 (5.6%)	33/215 (15.3%)	17/215 (7.9%)	-	-	-
Gandhi et al, 2003	43/181 (23.7%)	24/181 (13.2%)	7/181 (3.9%)	15/181 (8.3%)	-	-	7/181 (3.9%)
Roughead et al, 2004	72/186 (38.7%)	49/186 (26.3%)	-	25/186 (13.4%)	-	-	-
Nguyen et al, 2006^j^	12/53 (22.6%)	21/53 (39.6%)	8/53 (15.1%)	2/53 (3.8%)	1/53 (1.9%)		9/53 (17.0%)
Miller et al, 2013	166/912 (18.2%)	124/912 (13.6%)	44/912 (4.8%)	71/912 (7.8%)	32/912 (3.5%)	-	-
Marcum et al, 2013	41/57 (71.9%)	2/57 (3.5%)	-	-	-	-	14/57 (24.6%)
Median	27.3%	13.4%	4.8%	8.3%	2.7%	5.1%	12.7%
*All age group*							
Honigman et al, 2001^k^	89/121 (73.5%)	12/121 (9.9%)	10/121 (8.3%)	6/121 (4.9%)	6/121 (4.9%)	-	-
Gonzalez-Rubio et al, 2011	127/543 (23.4%)	126/543 (23.2%)	112/543 (20.6%)	71/543 (13.0%)	35/543 (6.4%)	22/543 (4.0%)	3/543 (0.2%)
Iancu et al, 2015	38/100 (38.0%)	-	-	-	-	-	-
Median	38.0%	16.5%	14.5%	8.9%	5.6%	4.0%	0.2%
*Paediatric*							
Horen et al, 2002	-	2/20 (10.0%)	14/20 (70.0%)	-	2/20 (10.0%)	1/20 (5.0%)	1/20 (5.0%)
Jonville-Bera et al, 2002	-	-	8/8 (100%)	-	-	-	-
Median		10.0%	85.0%		10.0%	5%	5%

Reported percentages do not always total 100% because several studies reported only the most common drug class associated with ADRs.

^a^Includes renin-angiotensin-aldosteron system (RAAS) agents (ACEIs and angiotensin receptor blockers (ARBs)) CCBs, beta-blockers, diuretics, lipid-lowering drugs, cardiac glycosides, anti-platelet, anti-coagulants.

^b^Includes antipsychotics, antidepressants, anticonvulsants/antiepileptics, analgesics, and opioids.

^c^Includes antibotics and vaccines.

^d^Includes NSAIDs, antirheumatic agents, muscle relaxant, and drugs for bone structures and mineralisation (e.g., bisphosphonates).

^e^Includes antihyperglycemic agents, drugs for peptic ulcer and gastro-oesophargeal reflux disease (GORD).

^f^Includes bronchodilators, mucolytics.

^g^Includes corticosteroids, drugs affecting endocrine system, and sex hormones.

^h^39 ADRs involves multiple drugs.

^i^Include cardiovascular and blood system drugs, including anticoagulants (n = 6) and hematinics (n = 5).

^j^Authors only reported medication implicated in the ADRs occurred in patients using ≥ 9 medication (n = 53).

^k^Authors reported 121 ADRs with several ADRs associated with more than one medication class category.

### Risk factors of ADRs

Multimorbidity condition was found to be a strong predictor of ADRs in the primary care, as well as the higher number of referrals to different specialties [48]. Number of medication prescribed was consistently reported as a major determinant of ADRs [23, 48]. Honigman et al showed that patients with ADRs were reported to take almost three times the number of drugs compared to those without ADRs [31]. Gandhi et al further demonstrated that the mean number of ADRs per patient was likely to be increased by 10% for one additional medication prescribed [23]. Other risk factors reported included the number of consultations to family physician, being female, off-label drug use, and exposure to several medication classes (i.e., antiinfectives and systemic hormonal preparation) [23, 26, 31, 39, 48] (Table 5).

**Table 5:** Risk factors of ADRs in the primary care setting.

Reference	Risk Factors	Method	Parameter	P-value
Gandhi et al [23]	Number of medications prescribed	Poisson regression	RR 1.1 (1.06, 1.15)	<0.001
Calderon-Larranaga et al [48]	Level of multimorbidity (moderate; high; very high)	Multivariable logistic regression	OR 4.24 (3.08, 5.85);	<0.001
			OR 17.58 (12.23, 25.26);	<0.001
			OR 45.26 (26.97, 75.95)	<0.001
	Number of visits to family physician		OR 1.013 (1.00, 1.02)	0.008
	Number of referral to different specialties		OR 1.19 (1.12, 1.28)	<0.001
	Polypharmacy (≥6 active substances)		OR 1.34 (1.11, 1.63)	0.003
	Sex: Female		OR 1.307 (1.11, 1.538)	0.001
Horen et al [26]	Off-label drug use	Multivariable logistic backward stepwise regression	OR 3.44 (1.26, 9.38)	NR*
	Exposure to antiinfective drugs		OR 3.06 (2.32, 8.11)	NR*
	Exposure to systemic hormonal drugs		OR 4.20 (1.08;16.40)	NR*

^a^The authors stated that these variables are significant without specifying significance level.

### Quality assessment

All of the included studies reported study design, methods to identify ADRs, and data sources. Individuals who identified ADRs, either researchers or clinicians, were described in all studies. The process of establishing causal relationship was reported in more than a third of the studies (n = 13) [23, 25–27, 31, 39–41, 43, 44, 47, 50, 58] with the majority having used a validated tool, i.e., Naranjo algorithm (n = 7) [27, 31, 39, 40, 43, 44, 47] or French causality method (n = 3) [25, 26, 58]. One study used criteria defined by the authors that considered three aspects; i) temporal relationship (timing) between the use of drug and the symptom; ii) whether the patient attributed the symptom to the drug; and iii) the strength of published data on the relationship between the symptom and the drug [23]. Four studies assessed the preventability [23, 27, 31, 43] and a third of the studies (n = 11) assessed the severity of ADRs [23, 26, 27, 31, 40, 43, 44, 47, 49, 56, 59].

## Discussion

To the best of our knowledge, this is the first systematic review to provide comprehensive information on the overall burden of ADRs focusing on primary care with quantitative assessment and evaluation of the quality of included studies. The pooled prevalence of ADRs in the primary care setting was 8.32% (95% CI, 7.82, 8.83). The percentage of preventable ADRs in primary care ranged from 12.35–37.96%, with the pooled estimate of 22.96% (95% CI, 7.82, 38.09). The prevalence estimates varied significantly according to age group, method of ADRs detection, setting, and sample size.

The lack of other reviews investigating ADRs in primary care hinders comparison to previous evidence. A previous scoping review found that the most common ADRs observed in this setting were dose-related and allergic reactions, while idiosyncratic reactions were not common [17]. Our review significantly extends this finding through the use of a thorough search strategy, evaluation of study quality, preventability and severity; and detailed heterogeneity analysis. Our prevalence estimate was slightly lower than the estimate reported by Tache et al which included a subset of six ambulatory-based studies performed before 2008 (8.32% vs 12.80%) [13]. The difference might result from different ADRs detection methods as all studies used combined medical record review and patients survey. In our subgroup analysis, studies using this method (n = 16) exhibited the highest estimate, with the prevalence of 19.92%, 95 CI, 16.11, 23.72. Compared to the previous systematic reviews of ADRs as causes of hospital admission, our estimate is higher [72, 73]. It has been estimated that the frequency of ADRs occurred in the primary care is likely to be higher due to inclusion of mild-moderate symptoms compared to the those requiring urgent medical care which possibly represents only the most severe reactions in the community [6, 30].

Our review suggests that about one fifth of ADRs in primary care were preventable (22.96%, 95% CI, 7.82, 38.09). This finding was comparable with two earlier ambulatory-based reviews showing that 16.5–21% of ADRs in this setting were preventable [13, 74]. The most frequently cited causes of preventable ADRs included failure to recognise previously documented allergic reaction to the causative drug, drug-drug interactions overlooked, and inappropriate selection of medication and/or dosage for patients’ clinical condition (e.g., comorbidity, age) [23, 27, 31, 43]. Several initiatives have been performed to potentially reduce preventable medication harm in the primary care setting, including pharmacists-led medication review [75–78], clinical decision support (CDS) embedded in information system [79, 80], educational intervention [81, 82], and nurse-led medication monitoring, particularly in long-term care facilities [83–85].

Inadequate monitoring was also reported as one of the major contributing factors in preventable ADRs [23, 27, 31, 43]. Nevertheless, such monitoring is often inadequate in the primary care [86]. A recent study undertaken in the UK primary care on ACEIs and ARBs users found that only one-tenth of these patients had guideline-recommended creatinine monitoring [87]. Another study involving 27,355 patients with hypertension, further demonstrated that those who received routine potassium monitoring were less-likely to experience serious hyperkalemia associated with spironolactone and ACEIs/ARBs [88]. Thus, strengthening drug monitoring is likely to generate tangible clinical benefits for patients.

Despite considerable variation on how each study defined severity, this review found that majority (76.0–96.3%) of ADRs occurred in the primary care setting were of moderate-low severity [23, 26, 27, 31, 40, 43, 44, 47, 49, 56, 59]. Nevertheless, it is worth noting that these reactions might not be minor for patients, as these reactions might affect their quality of life, medication adherence, and subsequent health service utilization [43, 89, 90]. In addition, changes in the treatment regimen were required in over half of the ADRs [23, 26, 27, 31, 43, 44, 49, 56, 59]. Patients with ADRs may be at increased risk of suboptimal therapeutic outcome due to prolonged discontinuation, limited treatment options, and potentially impaired adherence [91, 92], yet there is little clarity on further impact of ADRs on clinical outcomes. Further studies should investigate the consequences of ADRs on treatment pattern changes and their outcomes, as this information may help inform clinicians on the most appropriate intervention strategies following the reaction and provide thorough understanding on the burden of ADRs for patients and the health system.

It is not surprising that in our subgroup analysis, studies focusing on the elderly population (≥65 years) showed a higher prevalence of ADRs compared to other age groups (28.43%, 95% CI 18.65, 38.21; n = 10). Altered pharmacokinetics due to physiological impairment is largely unavoidable in this population, putting them at particularly higher risks of developing such reactions [93]. In addition, up to 44% of the elderly were exposed to polypharmacy (the use ≥ 5 medications) [94]. Onder et al showed that about a quarter of people living in the nursing homes (mean age 83,5 ± 9.3) used ≥ 10 medications (i.e., excessive polypharmacy) to manage their medical conditions [95]. We found 42.22% (95% CI 17.57, 66.88) of residents (age ≥ 65 years) in this setting potentially having experienced ADRs. As the world’s population is ageing, mitigation of ADRs among the elderly will become increasingly important.

Studies combining medical record/notes/medication review and patient survey resulted in the highest proportion of ADRs compared to other approaches (19.92%, 95% CI 16.11, 23.73). Medical record review alone might have limitation, owing to inadequate documentation [43, 44, 96]. Due to intermittent nature of health care contacts in primary care, it is possible that ADRs were not adequately recognised and/or communicated, thus, additional information received from patients might identify more ADRs than those captured in the medical record [41, 43, 49, 56, 59]. Jordan et al showed that nurse-led patient monitoring has been shown to be effective to improve recognition of ADRs. Timely identification of ADRs is important to further prevent a deterioration of patients’ condition which may result in unnecessary healthcare utilization [83–85].

Trigger-based record review has been increasingly used in various settings to facilitate more targeted and efficient identification of ADRs [29, 33, 97]. In this review, it generated comparable, but slightly lower estimates compared to manual chart review. Nevertheless, our result was derived from only limited studies (n = 3) that used the former method [30, 31]. In this approach, only records containing specific trigger indicators were further assessed, possibly limiting the capture of ADRs not associated with the pre-defined triggers. Several ADRs triggers with high-moderate positive predictive values (PPV) in primary care included INR >5, creatinine >2.5 mg/dL, thyroid stimulating hormone (TSH) <0.03 mLU/L for thyroxine, serum theophylline >20 microgram/mL, medication discontinued, and new order for ARBs [28, 30–32].

We found five studies using general practice database screening to identify readily-available ADRs data recorded by primary care providers during routine care [14, 42, 48, 61]. This approach reflects how primary care physicians recognise and document ADRs in a real-world setting, thus, the Hawthorne effect (i.e., observer effect) was likely to be minimal compared to a solicited reporting method [26, 58]. Nevertheless, differences in recording practice might hinder precise estimation [98]. Miguel et al demonstrated that a smaller prevalence of ADRs identified by administrative databases screening compared to manual chart review (2.4% versus 9.0%) was not a limitation, considering high PPV obtained (87.6%) and the reduced resource utilised (two person-hours versus 35 person-hours) [24].

There was considerable variation with regard to the risk factors of ADRs among the studies. Multimorbidity and referrals to different specialties were reported as significant predictors of ADRs [48]. A different result was observed by Tsang et al which showed that having one or more referrals was protective against adverse events [99]. Lack of coordination at different levels of care might put patients, particularly those with multimorbidity, at a higher risk of ADRs, due to the increased risk of potentially harmful drug-drug and/or drug-disease interactions, and non-adherence [100, 101].

Our finding showed that the most commonly implicated drugs in the ADRs in the primary care setting were cardiovascular drugs [23, 26, 31, 39, 40, 42, 45, 47, 49, 51, 58, 61, 63]. This is consistent with the existing evidence [13, 72]. Cardiovascular drugs, particularly RAAS agents, CCBs, lipid-modifying agents, and aspirin were found to be among the most frequently prescribed medications in primary care in the UK, US, and the Netherlands [102–105]. Thus, it is imperative for primary healthcare professionals to be vigilant in managing ADRs for this particular medication class [106–108].

Patient-provider awareness of relevant ADRs associated with patients’ medications and adequate patient-provider communication were important aspects in the management of ADRs in less-controlled healthcare environment such as primary care [14]. However, only about one-third of patients in the community had received information on ADRs [109, 110]. Healthcare professionals are often hesitant in giving information about important ADRs due to potential nocebo effects (i.e., perceived adverse effects as the result of negative expectancies) [111], nevertheless, a previous study showed the opposite, i.e, not receiving information on potential side effects from healthcare professional was associated with increased risk of self-reported ADRs and decreased satisfaction [43]. It is possible that patients who receive such information will better manage the drug reactions and become less worried [43, 112]. In specific therapeutic areas such as diabetes management, previous studies found that up to 48% patients were often uninformed about drug-induced hypoglycemia risk and thus unable to recognise this reaction [109, 110, 113]. This highlights the need for better education strategies by their primary care providers as the majority of patients with chronic diseases were routinely managed in the primary care setting [114].

### Implementation for practice and research

ADRs constitute a significant health problem in primary care, with about a fifth of ADRs identified as preventable. This indicates potential areas for improvement, particularly targeting errors in prescribing (contraindication, drug interactions, inappropriate selection of dosage/frequency for patients’ condition, previously documented drug allergy) and inadequate monitoring, particularly for patients with multimorbidity, advanced age, and concomitant use of medications. There is also a need to improve patient-provider communication of ADRs to prevent further iatrogenic complication and unnecessary healthcare utilisation. Weingart et al showed that an electronic patient-centered portal, enabled patients to ask question and report problem about their prescribed medication, was effective in improving communication about medication problems and was able to identify ADRs in the primary care setting [115, 116]. In addition, further educational support for both patient and provider may be beneficial to increase general awareness on the safe use of medicines and improve safety culture [23, 117, 118].

Current knowledge of ADRs has focused on the frequency, with only limited studies reflecting how ADRs impact patient’ health status. Although most of the ADRs in the primary care setting are not likely to pose life-threatening condition for patients, the consequences on health-related outcomes might be significant. It could interfere with patient treatments and result in suboptimal therapeutic outcomes, yet there is little clarity about the impact of ADRs on treatment pattern changes and its associated outcomes, particularly for high-risk therapeutic area [91, 92]. Such information would allow identification of appropriate strategies following the ADRs which best fit patients’ circumstances and provide thorough understanding on the burden of ADRs for patients and the health system.

### Strengths

The main strength of this review is that this is the first systematic review with quantitative assessment and heterogeneity analysis on the burden of ADRs in the primary care with evaluation of the quality of the studies. We presents detailed information on factors contributing to heterogeneity, preventability, medication class frequently implicated, severity, and risk factors of ADRs. In addition, the risk of bias of included studies were assessed using the specific assessment instrument for ADRs studies.

### Limitations

The finding of this review should be interpreted in light of its limitations. Firstly, there was a substantial heterogeneity in the reported prevalence between studies. Previous systematic review showed that high statistical heterogeneity is more frequent in meta-analyses of prevalence compared to binary outcome [115, 119]. We performed subgroup analysis and meta-regression to allow better identification of potential source of variability, showing that different ADRs detection methods, age group, setting, and sample size affected the estimates. Secondly, there was no uniformity with regard to description of medications associated with ADRs. Some studies described the medication in Anatomical Therapeutic Chemical (ATC) level and others in specific drug class/active substances level, making the comparison challenging. Thirdly, all eligible studies were performed in the context of European, North America, and Australian healthcare systems, which limit the generalisability of the results. Nevertheless, the finding of this review might serves as basis estimate for other countries, where the prevalence of overall ADRs in primary care have yet to be characterised.

## Conclusion

ADRs constitute a significant health problem in the primary care setting. Cardiovascular system drugs were the most commonly implicated medication class. Further research should focus on examining whether ADRs affect subsequent clinical outcomes, particularly in high-risk therapeutic areas. Such understanding might better inform strategies to reduce the burden of ADRs in the primary care setting.

## Supporting information

S1 Appendix PRISMA 2009 checklist. (DOC)

S2 Appendix Search strategy. (DOCX)
