# Supplementary material for: Prevalence of adverse drug reactions in the primary care setting: A systematic review and meta-analysis
Source: PLoS One. 2021 May 26;16(5):e0252161. doi: 10.1371/journal.pone.0252161 (PMC8153435; doi:10.1371/journal.pone.0252161)
Supplement: S2 Appendix — (DOCX) [file pone.0252161.s002.docx]

# S2 Appendix. Search Strategy

1. "Drug-Related Side Effects and Adverse Reactions"/ [MeSH term]
2. "Drug Therapy/Adverse Effect"/ [MeSH term]
3. "Adverse Drug Reaction*".ti,ab.
4. "Adverse Drug Event*".ti,ab.
5. "Drug Side Effect*".ti,ab.
6. (Drug or Medicine or Medication) adj2 "related problem"
7. 1 or 2 or 3 or 4 or 5 or 6
8. "Primary Health Care"/ [MeSH term]
9. "Primary care".ti,ab
10. "Primary Medical Care".ti,ab.
11. "General Practice"/ [MeSH term]
12. "General Practi*".ti,ab.
13. "Family Practice"/ [MeSH term]
14. "Family Practi*"ti,ab.
15. "Community Health Services"/ [MeSH term]
16. "Community Pharmac*".ti,ab.
17. "Ambulatory Care"/ [MeSH term]
18. "Ambulatory Care".ti,ab.
19. Outpatients/ [MeSH term]
20. "Outpatient*”.ti,ab.
21. “Nursing Homes”/ [MeSH term]
22. “Nursing Home*”.ti,ab.
23. “Long-term Care”/ [MeSH term]
24. “Care Home*”.ti,ab.
25. “Home Care Services”/ [MeSH term]
26. “Home Care Service*”.ti,ab.
27. 8 or 9 or 10 or 11 or 12 or 13 or 14 or 15 or 16 or 17 or 18 or 19 or 20 or 21 or 22 or 23 or 24 or 25 or 26
28. 7 and 27
